# Electrochemical Biosensors Based on Ferroceneboronic Acid and Its Derivatives: A Review

**DOI:** 10.3390/bios4030243

**Published:** 2014-07-30

**Authors:** Baozhen Wang, Shigehiro Takahashi, Xiaoyan Du, Jun-ichi Anzai

**Affiliations:** 1 Department of Nutrition and Food Hygiene, School of Public Health, Shandong University, 44 Wenhua Xilu, Jinan, Shandong 250012, China; E-Mail: bzhenw@hotmail.com; 2 Graduate School of Pharmaceutical Sciences, Tohoku University, Aramaki, Aoba-ku, Sendai 980-8578, Japan; E-Mail: t-shigehiro@m.tohoku.ac.jp; 3 Public Health College, Harbin Medical University, Harbin 150081, China; E-Mail: duxiaoyanha@163.com

**Keywords:** ferroceneboronic acid, ferrocene-modified boronic acid, electrochemical sensor, glucose sensor, HbA1c sensor, F^−^-ion sensor

## Abstract

We review recent progress in the development of electrochemical biosensors based on ferroceneboronic acid (FcBA) and ferrocene (Fc)-modified boronic acids. These compounds can be used to construct electrochemical biosensors because they consist of a binding site (*i.e.*, a boronic acid moiety) and an electrochemically active part (*i.e*., an Fc residue). By taking advantage of the unique properties of FcBA and its derivatives, electrochemical sensors sensitive to sugars, glycated hemoglobin (HbA1c), fluoride (F^−^) ions, and so forth have been widely studied. FcBA-based sugar sensors rely on the selective binding of FcBA to 1,2- or 1,3-diol residues of sugars through the formation of cyclic boronate ester bonds. The redox properties of FcBA-sugar adduct differ from those of free FcBA, which forms the basis of the electrochemical determination of sugars. Thus, non-enzymatic glucose sensors are now being actively studied using FcBA and Fc-modified boronic acids as redox markers. Using a similar principle, HbA1c can be detected by FcBA-based electrochemical systems because it contains hydrocarbon chains on the polypeptide chain. HbA1c sensors are useful for monitoring blood glucose levels over the preceding 8–12 weeks. In addition, FcBA and Fc-modified boronic acids have been used for the detection of F^−^ ions due to the selective binding of boronic acid to F^−^ ions. F^−^-ion sensors may be useful alternatives to conventional ion-selective electrodes sensitive to F^−^ ion. Furthermore, FcBA derivatives have been studied to construct lectin; steroids; nucleotides; salicylic acid; and bacteria sensors. One of the limitations of FcBA-based sensors comes from the fact that FcBA derivatives are added in sample solutions as reagents. FcBA derivatives should be immobilized on the surface of electrodes for developing reagentless sensors.

## 1. Introduction

Electrochemical biosensors are constructed by modifying the surface of metal and carbon electrodes with biomaterials such as enzymes, antibodies, and DNA. The output signal of biosensors is generated through the specific binding or catalytic reactions of the biomaterials on the electrode surface. A characteristic feature of biosensors arises from the highly selective reactions of the biomaterials to the target compounds. For instance, glucose oxidase (GOx)-based biosensors are used for the specific determination of blood glucose among possible interfering components in samples. Thus, glucose biosensors are currently widespread in clinical laboratories and hospitals for monitoring the blood glucose levels of diabetic patients [1]. However, enzyme biosensors often suffer from intrinsic drawbacks, including instability and inhomogeneity of enzyme activity. Thus, calibration of the biosensor response immediately before use is essential. Low durability and the relatively high cost of enzymes also limit the use of biosensors. Much effort has therefore been devoted to the development of non-enzymatic biosensors using synthetic materials in place of proteins. 

**Figure 1:**
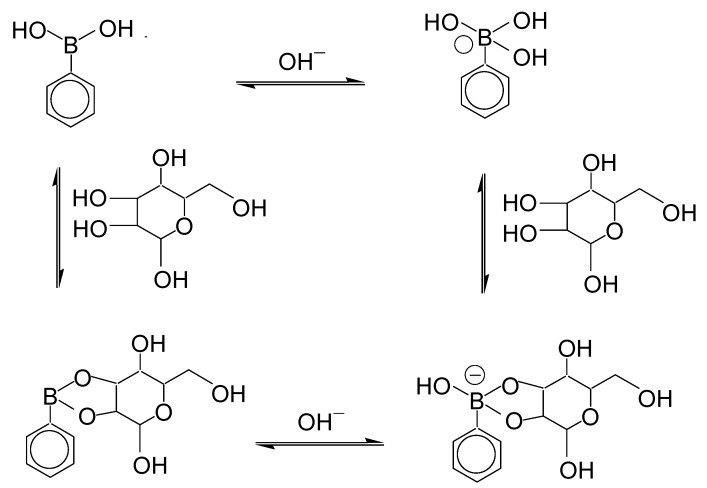
Binding equilibria of phenylboronic acid to sugar and OH^−^ ion.

In this review, we focus on the development of electrochemical sensors based on ferroceneboronic acid (FcBA) and Fc-modified boronic acids, which are mainly used for detecting sugars and related compounds due to the specific recognition of 1,2- and 1,3-diol residues in sugars by boronic acid residues [2]. In the binding equilibria of phenylboronic acid (PBA) to sugar and OH^−^ ion (Figure 1), PBA forms a negatively charged species in basic media characterized by an electron-rich sp^3^ boron atom with tetrahedral geometry, which in most cases shows higher binding affinity to sugars than the non-ionic form. PBA binds to sugar, forming a boronate ester, and becomes negatively charged, although free PBA is neutral at the same pH because the p*K*_a_ of the boronate ester is usually lower than that of the parent PBA (see Figure 1). The change in the electrical state of the boronic acid moiety would induce changes in the optical and redox properties of the compounds. Thus, it is reasonable to assume that the redox properties of the Fc residue of FcBA and Fc-modified boronic acids may be altered upon sugar binding. The present review provides an overview of electrochemical biosensors developed using FcBA and Fc-modified boronic acids. It should be noted here that FcBA and derivatives are used in most cases as redox-active reagents dissolved in sample solutions without being immobilized on the surface of electrodes. Therefore, the development of FcBA-modified electrodes as reagentless sensors is still in its infancy. Importance of developing reagentless sensors has recently been discussed by Wolfbeis [3].

## 2. Sugar Sensors

**Figure 2:**
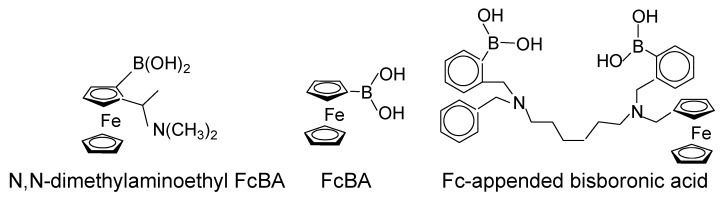
Chemical structures of ferroceneboronic acid and its derivatives.

The potential use of FcBA derivatives for the electrochemical determination of sugar was first demonstrated by Ori and Shinkai nearly 20 years ago [4]. They prepared *N*,*N*-dimethylaminoethyl-substituted FcBA (Figure 2) and recorded normal pulse voltammogram (NPV) in the absence and presence of sugars, including fructose, glucose, mannitol, and sorbitol. The reduction potential of the FcBA derivative shifted by *ca.* 50 mV in the negative direction in the presence of sugars at pH 7.0. The binding constants of the FcBA derivative to sugars based on the NPV results were evaluated and found to be significantly higher for ferrocenium ion (the oxidized form) than for its reduced form (non-ionic form). The results were explained by the higher acidity of the boronic acid residue in the oxidized form. An X-ray crystallographic study by Norrild and Søtofte revealed that intramolecular B-N bond is not available in *N*,*N*-dimethylaminoethyl-substituted FcBA [5]. On the other hand, Moore and Wayner studied the cyclic voltammetric (CV) behavior of FcBA (Figure 2) in aqueous solutions at pH 4.0–12 [6]. CV results showed that FcBA forms adducts with sugars in solution at pH 7.0. The kinetic and thermodynamic constants for adduct formation were determined from the CV results. The binding constants for the ferrocenium form of FcBA were about two orders of magnitude higher than those for the non-ionic form. Moore and Wayner suggested that the redox-dependent switching of the sugar binding could be exploited in the development of electrochemical sugar sensors. 

A series of Fc-modified boronic acids were synthesized to study the effects of an intramolecular B-N bonding motif on glucose binding [7]. Nuclear magnetic resonance and X-ray crystallographic studies revealed the existence of intramolecular B–N bonds in the solid phase. James and coworkers synthesized Fc-appended bisboronic acid (Figure 2) for the electrochemical sensing of glucose [8]. Differential pulse voltammogram (DPV) of bisboronic acid exhibited an oxidation peak at 0.36 V in the absence of glucose, while the peak shifted to 0.45 V upon the addition of 10 mM glucose. The above studies using FcBA and Fc-modified boronic acids suggested the potential use of these compounds in constructing electrochemical glucose biosensors.

**Figure 3:**
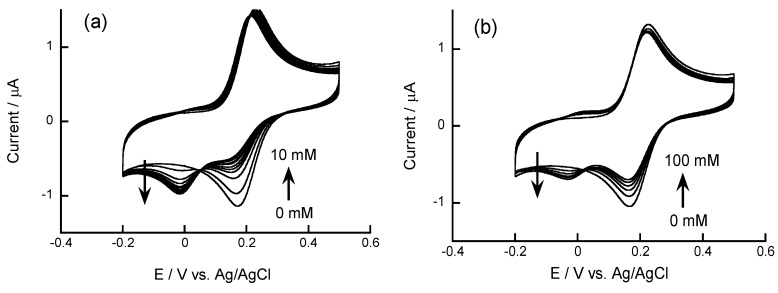
Cyclic voltammograms of 0.1 mM FcBA in the presence of (**a**) fructose and (**b**) glucose at pH 7.0. Scan rate: 50 mV·s^−1^. Reprinted with permission from Takahashi *et al*. [9]. Copyright (2011) Elsevier.

**Figure 4:**
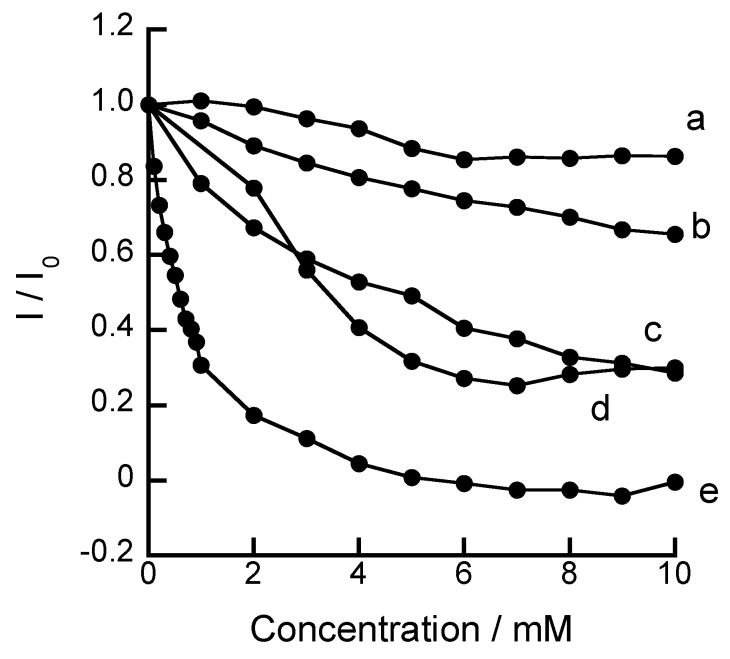
Changes in the reduction peak currents (I/I_0_) in CV of FcBA as a function of the concentration of phenolic compounds: (**a**) mandelic acid, (**b**) salicylamide, (**c**) 2-hydroxybenzylalcohol, (**d**) salicylic acid, and (**e**) salicylhydroxamic acid. Reprinted with permission from Takahashi *et al*. [9]. Copyright (2011) Elsevier.

Anzai and coworkers recently studied the voltammetric responses of FcBA to sugars, nucleosides, and phenolic compounds in aqueous solutions [9]. FcBA exhibited a pair of oxidation and reduction peaks in the CV at 230 and 170 mV *vs*. Ag/AgCl at pH 7.0, respectively, while another pair of redox peaks was observed in the presence of sugars (Figure 3). The changes in the redox peak currents depended on the concentration of the compounds, demonstrating the usefulness of FcBA in the voltammetric determination of these compounds. For instance, the reduction peak decreased in the presence of 1−50 mM of glucose and 1−10 mM of fructose. Among the phenolic compounds tested, FcBA exhibited the highest response to salicylhydroxamic acid (Figure 4). In addition, FcBA responded to cytidine and uridine, whereas the responses to 2*'*-deoxycytidine and 2*'*-deoxyuridine were negligible, suggesting that FcBA bound to the nucleosides through boronate ester formation with 2,3-diol units at the ribose moiety in the nucleosides. 

On the other hand, Lacina and Skládal aimed to immobilize FcBA on the surface of modified electrodes [10]. To achieve this goal, they used electrodes modified with sorbitol and 1,6-hexanediol. FcBA was immobilized to the surface of the electrode through boronate ester bonds. Thus, FcBA-modified electrodes may be useful as reagent-free sensors for the amperometric or voltammetric determination of sugars and related compounds. One problem that needs to be addressed is the low level of selective binding between FcBA and glucose. FcBA shows higher binding affinity to fructose than to glucose, which is the general trend for all boronic acids including PBA [2]. In this context, glucose selectivity could be improved by using boronic acid dimers [8,11]. 

FcBA can be used for the electrochemical probing of the isomerization of glucose to fructose because the redox potential of FcBA-fructose adduct is different from that of the FcBA-glucose composite. Liu and coworkers successfully monitored the isomerization reaction of glucose catalyzed by glucose isomerase under different reaction conditions [12]. They suggested that this sensor system may be useful in the food industry. The same group also monitored the catalytic activities of phosphoglucose isomerase and alkaline phosphatase from the voltammetric responses of FcBA in the presence and absence of enzyme inhibitors [13]. 

**Figure 5:**
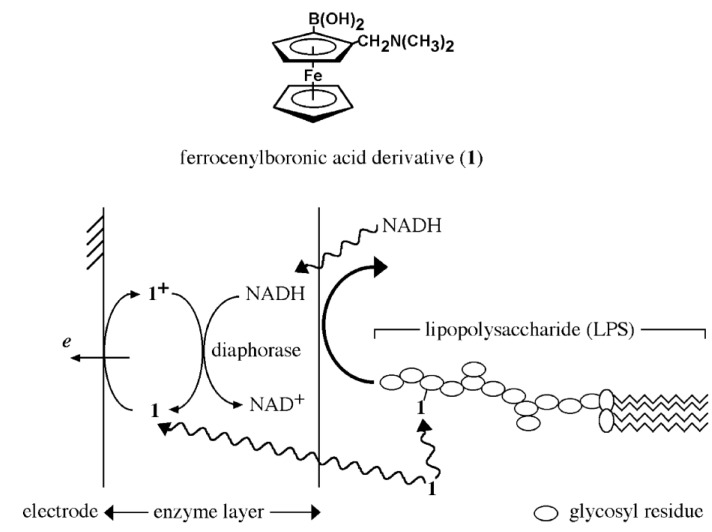
Chemical structure of *N*,*N*-dimethylaminomethyl-substituted FcBA (**1**) and a schematic illustration of reaction of LPS sensing by enzyme-modified electrode. Reprinted with permission from Kato *et al*. [14]. Copyright (2007) Elsevier.

Niwa and coworkers studied the amperometric determination of lipopolysaccharide (LPS) using a diaphorase-modified electrode in the presence of *N*,*N*-dimethylaminomethyl-substituted FcBA [14]. The FcBA derivative served as an electron transfer mediator between the enzyme and electrode to enhance the output signal. The magnitude of the output current depended on the concentration of LPS in the sample solution because LPS-bound FcBA marginally diffuses into the enzyme layer (Figure 5). The lower detection limit of LPS is reported to be 50 ng·mL^−1^.

**Figure 6:**
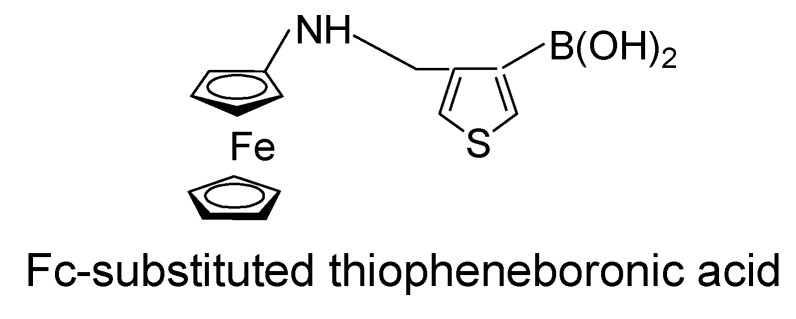
Chemical structure of Fc-substituted thiopheneboronic acid.

Lacina and coworkers have recently reported the synthesis and redox properties of Fc-substituted thiopheneboronic acid (FcTBA, Figure 6) [15]. FcTBA exhibited two discrete cathodic peaks associated with free and sugar-bound FcTBA in CV. FcTBA was immobilized on the surface of an Au electrode through Au-S bonds to construct FcTBA-modified sensors. Unfortunately, however, the binding of FcTBA to sugar was not strong enough, probably due to a lack of mobility and the rotational ability of FcTBA on the surface. Nevertheless, the FcTBA-modified electrode is a highly promising prototype for reagentless glucose biosensors.

## 3. HbA1c Sensors

Glycated hemoglobin (HbA1c) is a hemoglobin derivative formed by the non-enzymatic glycosylation of the hemoglobin β-chain. The percentage of HbA1c in total hemoglobin is known to reflect the glycemic status of diabetics over the preceding 8−12 weeks; the normal level of HbA1c is 4−6%. Therefore, HbA1c level is currently an important measure in the management of long-term glucose levels in blood. HbA1c levels can be determined by a variety of clinical procedures, including chromatography and spectroscopy [16,17]. HbA1c determination, based on these techniques, has recently been reviewed by Pundir and Chawla [18].

HbA1c can be determined by FcBA and its derivatives because it contains a glycosylated polypeptide chain. FcBA and derivatives bind to HbA1c and thus discriminate it from non-glycosylated hemoglobin. Several groups have studied the electrochemical determination of HbA1c levels based on FcBA and its derivatives. Scheller and coworkers reported on FcBA-based HbA1c sensors [19,20,21].

**Figure 7:**
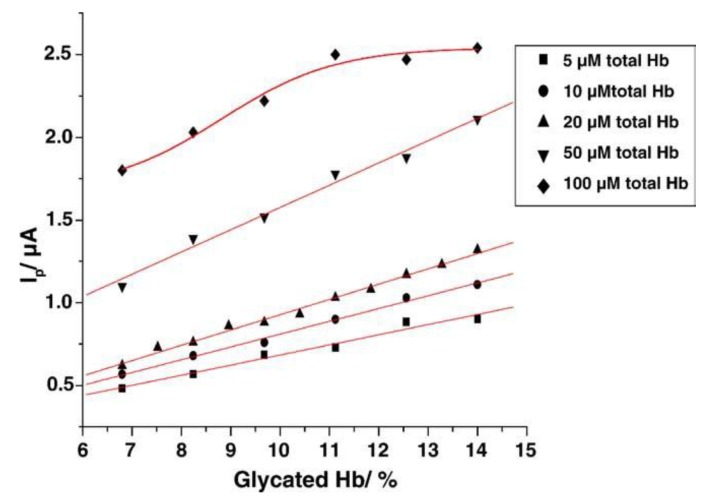
Calibration graphs for HbA1c in samples containing different total concentrations of Hb. The voltammetric peak current was recorded for 3 μL sample solutions. Reprinted with permission from Scheller *et al*. [19]. Copyright (2006) Elsevier.

They used sensor electrodes modified with zirconium dioxide nanoparticles to immobilize HbA1c from whole blood samples [19]. The sensor was incubated in FcBA solution to form HbA1c-FcBA complexes on the electrode surface. The peak current of the bound FcBA was linearly dependent on the percentage of HbA1c in the samples over the range 6.8–14% (Figure 7). Piezoelectric sensors were also used to detect HbA1c [20]. The surface of the piezoelectric quartz crystals underwent initial covalent modification with deoxycholic acid, and the probe was incubated in sample solution containing preformed HbA1c-FcBA adducts. In this procedure, the amount of HbA1c is estimated from the redox current of FcBA while the total hemoglobin (*i.e*., HbA1c + non-glycosylated hemoglobin) can be deduced by measuring the change in resonance frequency of the sensor. This sensor can be used to determine HbA1c in the range 1–20%. The sensor probe can be used repeatedly more than 30 times after regeneration of the sensor surface by pepsin digestion. A further improvement in HbA1c sensors was reported by the same group, in which the piezoelectric sensors were combined with anti-HbA1c antibody [21]. The sensor probe is incubated in the HbA1c sample solution, followed by measurement of the resonance frequency of the sensor to evaluate the total amounts of glycated and non-glycated hemoglobin adsorbed. The HbA1c on the sensor surface is recognized by anti-HbA1c antibody. The antibody can be modified with FcBA because immunoglobulin molecules contain hydrocarbon chains. The sensitivity of this sensor is threefold that of the FcBA-based HbA1c sensors without the antibody. 

**Figure 8:**
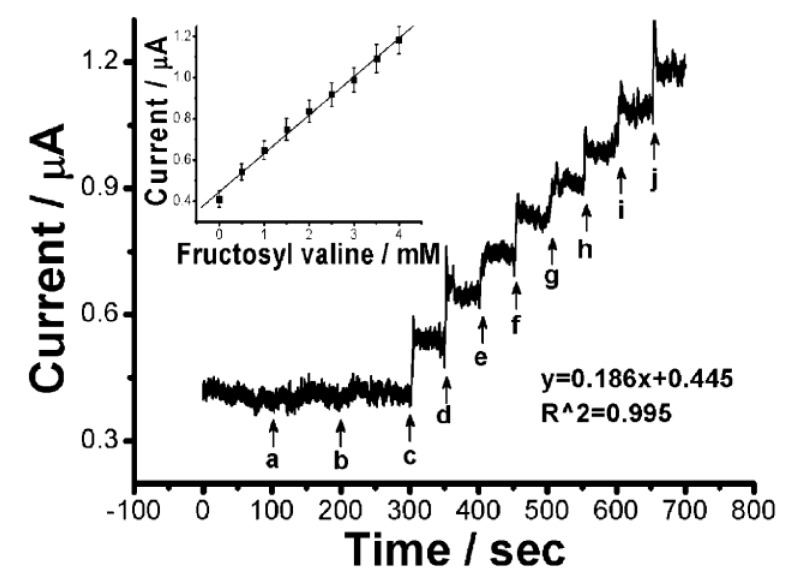
Amperometric response of FcBA to glucose and FV. The output current was recorded at 0.1 V *vs*. Ag/AgCl. 1 mM glucose (**a**,**b**) and 0.5 mM FV (**c**–**j**) were added to the sample solution. (Inset) A calibration graph for FV. Reprinted with permission from Chien *et al*. [24]. Copyright (2011) Wiley-VCH Verlag GmbH & Co.

Other groups have reported boronic acid-modified sensors combined with FcBA or Fc for the electrochemical determination of HbA1c [22,23,24]. Song and Yoon used dendrimer- and GOx-modified sensors to enhance the output signals of the sensors in which FcBA or Fc was used as an electron transfer mediator [22,23]. On the other hand, Chien and Chou reported amperometric sensors for fructosyl valine (FV) based on FcBA [24]. FV is released from HbA1c by protease digestion, enabling an indirect estimation of HbA1c levels. FV-FcBA adducts exhibited reduction potential at more cathodic regions compared with free FcBA, allowing FV to be electrochemically determined. The amperometric signal linearly depended on the concentration of FV of mM level whereas the response to glucose was negligible (Figure 8). The same authors tried to determine FV through direct oxidation on a glassy carbon paste electrode, in which the electrode potential of 1.0 V was applied [25]. In contrast, FV can be detected 0.1 V by using FcBA, showing an advantage of using FcBA. In this context, it is worth noting that several papers have reported enzyme biosensors for the detection of FV [26,27,28,29]. Flow-injection sensors [26], disposable thick film sensors [27], and core-shell nanoparticle-modified electrodes [28] have been reported for FV determination. In addition, various types of HbA1c sensors have been developed based on surface plasmon resonance [29], impedance spectroscopy [30,31], field effect transistors [32,33], molecular imprinted polymers [34,35,36], and quartz-crystal microbalance [37]. 

## 4. Fluoride Ion Sensors

The monitoring of fluoride ion in tap water is a crucial issue for improving healthcare and environmental issues [38]. Shinkai and coworkers reported the selective determination of fluoride (F^−^) ion based on changes in the redox property of FcBA [39]. The redox potential of FcBA shifted linearly in a negative direction depending on the logarithm of the F^-^ concentration, log [F^−^], over the range 1 mM to 3 M. This potential change was highly selective towards F^−^ ion compared with other halogen ions, such as chloride and bromide. These results can be explained by the F^−^ ion binding to the boron atom as a strong Lewis acid. The selectivity of this system to F^−^ ion is comparable to that of conventional F^−^ ion-selective electrodes, which rely on changes in the electrode potential across a LaF_3_ membrane. The same group further investigated the F^−^ ion sensing system by redox dye coupling [40]. They found that the UV absorption intensity of redox dye (methylene blue, MB) at 665 nm decreased in response to F^−^ ion, because of the reduction of MB by F^−^ ion-bound FcBA. The redox reaction between MB and FcBA was highly dependent on the concentration of F^−^ ion, enabling colorimetric determination by the naked eye. F^−^ ion-dependent changes in the redox potential of FcBA are responsible for the color changes of MB. 

**Figure 9:**
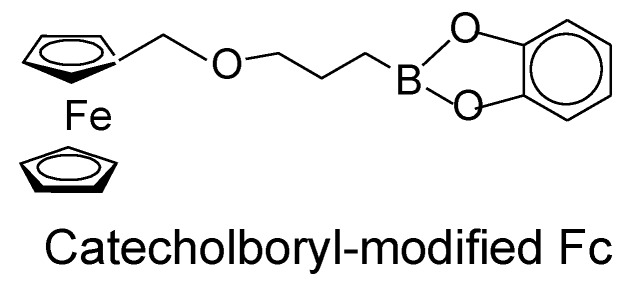
Chemical structure of catecholboryl-modified ferrocene.

The Fallis and Aldridge group studied F^−^ ion binding and redox properties of Fc derivatives with mono-, bis-, and tetra-boronate esters [41,42]. If the shift in redox potential of the Fc derivative induced upon F^−^ ion binding is sufficiently large, the Fc moiety is aerobically oxidized. In such a case, F^−^ ion- may be detected by a color change in the Fc derivatives because their oxidized form exhibits a new absorption band around 600 nm, which is associated with a charge-transfer process. In practice, an F^−^ ion-induced redox potential shift for Fc derivative with mono-boronate ester was insufficient to induce a color change, while Fc derivatives with multiple boronate esters produced color changes upon the addition of F^-^ ion owing to large shifts in the redox potential. Ghosh and coworkers have recently synthesized catecholboryl-modified Fc (Figure 9) and studied the electrochemical and spectroscopic properties [43]. The compounds exhibited a large cathodic shift in redox potential upon binding F^−^ ion, followed by colorimetric changes associated with the aerobic oxidation of Fc moiety of the compounds. It is worth mentioning that F^−^ ion-induced shifts in the redox potential of the Fc derivatives depend significantly on the type of solvents: redox and colorimetric responses in water are usually weak.

## 5. Miscellaneous

FcBA binds to nucleosides by forming boronate ester bonds with a ribose moiety. The Xia and Li group constructed label-free electrochemical sensors sensitive to oligomers of ribonucleic acid (RNA) using FcBA as a redox marker [44]. The surface of an Au electrode was modified with a monomolecular layer of single-stranded deoxyribonucleic acid (DNA) composed of 21 complementary bases for the target RNA. After the electrode was incubated in the sample solution, FcBA was attached to the ribose moiety of the hybridized RNA. Thus, hybridized RNA can be electrochemically detected by voltammetric measurement of FcBA. One advantage of FcBA is that only the RNA chain is recognized by FcBA because 1,2-diol functionality is not available to DNA. The sensor can be used to detect complementary 21-mer RNA chains in the concentration range 5 nM to 1 μM. 

Gamoh and coworkers used FcBA to label steroids with the 1,2-diol moiety for HPLC analysis with electrochemical detection [45]. They demonstrated the usefulness of FcBA labeling by verifying several brassinosteroids in sunflower pollen, including brassinolide, dolichosterone, norcastasterone, and castasterone. The lower detection limit for HPLC analysis of brassinosteroids was at the 10^−12^ g level. Du and coworkers reported a protocol for the determination of catechin and protocatecuic acid based on DPV measurements using FcBA [46]. DPV measurements provided useful calibration graphs in the catechin concentration range 0.5–4.0 mM and the protocatechuic acid range 1–12 μM. 

The catalytic activity of tyrosinase can be evaluated using tyramine-modified electrodes coupled with FcBA [47]. Tyrosinase oxidizes the phenolic residue of tyramine to form catechol functionality on the surface of the electrodes. The resulting catechol group was labeled with FcBA, providing a readout signal for the catalytic activity of the enzyme. Thus, the enzymatic reaction of tyrosinase can be monitored from the redox signal of FcBA. Furthermore, this technique can be used to evaluate the catalytic activity of proteinases by using electrodes modified with catechol-terminated peptide oligomers. After FcBA labeling, the modified electrode is treated with a proteinase such as thrombin to hydrolyze the peptide chain. The intensity of the redox signal arising from FcBA decreases with time, depending on the catalytic activity of the enzyme.

It has been reported that boronic acids bind not only to 1,2- and 1,3-diol compounds but also to α- and β-hydroxy carboxylic acids to form boronate esters [48]. In this context, Anzai and coworkers studied the voltammetric response of salicylic acid (SA) and its derivatives using FcBA [49]. The reduction peak of FcBA was observed at 200 mV *vs*. Ag/AgCl in DPV at pH 7.0 without SA, while the peak current decreased and a new reduction peak appeared at 50 mV in the presence of SA depending on the concentration. The detection range for SA was at a concentration of 1–10 mM. 

**Figure 10:**
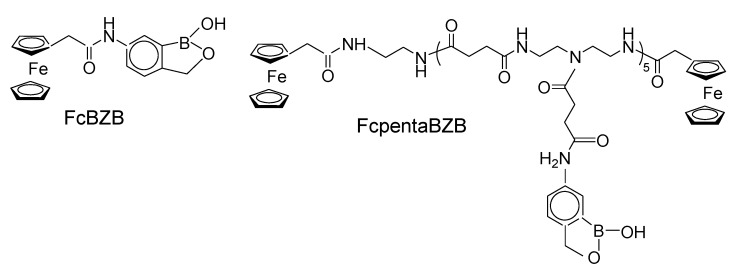
Chemical structures of ferrocene-modified benzoboroxoles.

Electrochemical displacement sensors sensitive to lectin and *E. coli* were fabricated by the Gajovic-Eichelmann and Scheller groups using FcBA and ferrocene benzoboroxoles (FcBZB, FcpentaBZB) (Figure 10) [50]. The surface of an Au electrode was modified with a mannose monolayer and either FcBA or FcBZBs was assembled as a redox-active marker onto the mannose-modified surface. The marker molecules were displaced upon exposing the electrode to lectin (Con A) and removed from the surface, decreasing the redox current of the marker. Thus, Con A could be determined over the concentration range 38 nM to 5.76 μM. The sensors operate even at pH 7.4 because the FcBZB derivatives bind to the electrode surface at neutral pH. Sensor response to non-glycated proteins such as bovine serum albumin and ribonuclease A was lower than that to Con A. FcpentaBZB exhibited the highest response among the marker molecules used. The sensor was also used to detect *E. coli* with a detection limit of 600 cells·mL^−^^1^. These researchers suggested that this sensor would be useful for detecting other carbohydrate-binding proteins, such as toxic plant lectins and other pathogens, by modifying the sensor surface with suitable carbohydrates. 

Tucker and coworkers synthesized a PBA derivative bearing a chiral Fc moiety and determined the enantiomeric excess of bisnaphthol based on the shift of the redox potential [51]. (R)-2a/(R)-bisnaphthol adduct showed redox potential at 665 mV (*vs*. decamethylferrocene as the internal reference) while the redox potential was observed at 614 mV for (R)-2a/(S)-bisnaphthol adduct. The redox potential depended linearly on the percentage of enantiomeric excess of bisnaphthol. Gamoh and coworkers also studied the use of chiral FcBA for the optical resolution of enantiomeric diols [52]. FcBA derivatives containing chiral centers appear promising in the development of chiral selective sugar sensors.

## 6. Conclusions

FcBA and Fc-modified boronic acids have been used for the development of electrochemical biosensors by taking advantage of the selective binding of boronic acids to 1,2- and 1,3-diol compounds. FcBA and derivatives are used for the voltammetric determination of glucose because the redox potential or redox current is dependent on the concentration of the glucose. In a similar way, HbA1c can be determined using FcBA to monitor the glycemic status of diabetics over the preceding 8–12 weeks. FcBA and its derivatives are also useful for detecting catechols and related compounds in addition to fluoride ions. They are usually added into the sample solutions. Sensor systems could be further improved by immobilization of FcBA and its derivatives on the surface of electrodes without loss of redox and binding abilities. For this goal, FcBA derivatives substituted with thiol end groups would be useful because thiol groups easily bind to Au electrode surface to form monomolecular layer. FcBA-bearing polymers may be also useful to form thin films on the electrode surface.
